# Changing values of farm animal genomic resources. from historical breeds to the Nagoya Protocol

**DOI:** 10.3389/fgene.2015.00279

**Published:** 2015-09-08

**Authors:** Sakari Tamminen

**Affiliations:** Department of Social Research, University of Helsinki, Helsinki, Finland

**Keywords:** values, animal genetic resources, convention on biological diversity, interlaken declaration, global plan of action, national genetic landscapes

## Abstract

The paper reviews the history of Animal genetic resources (AnGRs) and claims that over the course of history they have been conceptually transformed from economic, ecologic and scientific life forms into political objects, reflecting in the way in which any valuation of AnGRs is today inherently imbued with national politics and its values enacted by legally binding global conventions. Historically, the first calls to conservation were based on the economic, ecological and scientific values of the AnGR. While the historical arguments are valid and still commonly proposed values for conservation, the AnGR have become highly politicized since the adoption of the Convention of Biological Diversity (CBD), the subsequent Interlaken Declaration, the Global Plan for Action (GPA) and the Nagoya Protocol. The scientific and political definitions of the AnGRs were creatively reshuffled within these documents and the key criteria by which they are now identified and valued today were essentially redefined. The criteria of “*in situ* condition” has become the necessary starting point for all valuation efforts of AnGRs, effectively transforming their previous nature as natural property and global genetic commons into objects of national concern pertaining to territorially discrete national genetic landscapes, regulated by the sovereign powers of the parties to the global conventions.

## Animal Genetic Resources as a Global Matter of Concern

Animal genetic resources (AnGRs) have become a topic of renewed interest in international politics taking agricultural species as its object of concern for the last decade. The reasons for this point to a number of intertwined reasons. The first has to do with the unclear legal status and the scope of regulation stemming from biodiversity agreements targeting a wide range of animal species from wild to agricultural, their biological materials and genetic resources within the global politics of late 20th and early 21st century. Here, states, nations and indigenous communities have become new key stakeholders of genetic resources as they have been granted sovereign rights over territorially bound, native “*in situ*” resources within the text of the convention on biological diversity (CBD)^1^, signed by over 150 states in 1992. The sovereign rights over GRs have been subsequently re-enforced with the adoption of the Nagoya Protocol on Access and Benefit-sharing^2^ entering into force on October 2014. The Nagoya Protocol is the legally binding protocol guiding how to interpret and act upon the genetic resources issues presented in the CBD over 20 years earlier.

The second, and inherently nested interest relates to the way in which access and animal could, or should, be regulated internationally under biodiversity frameworks, including debates on how different kinds of genetic resources from plants to animals and from wild to agricultural species differ from each other and how the difference might have to inform the practical execution of their global governance. The Food and Agriculture Organization of the United Nations (FAO) established a Commission on Plant Genetic Resources in 1983 to deal with policy, access and benefit sharing issues related plant genetic resources. FAO did broaden the mandate of the Commission in 1995 to cover all aspects after the CBD entered into force and after recognizing “that broadening the coverage of the Commission would allow the Organization to deal in a more integrated manner with agrobiodiversity issues” (Food and Agriculture Organization, 1995, p. 66). 2 years later in 1997, the Commission also established separate working groups for animal and plant genetic resources, followed by one expert group for forest genetic resources^3^. All these Committees—specifically established for different types of genetic resources—demonstrate how difficult the policy, ownership and access and benefit sharing issues related to GRs, and especially to AnGRs, are to understand, let alone to manage in practice.

Three examples from international analyses from the last 10 years will clarify some of the difficult aspects related to global agreements and governance on agricultural AnGRs and open up good questions how did the AnGRs become so politically contested objects of agricultural nature.

Consider, for example, a report from 2006 exploring policy options for the “Exchange, Use and Conservation of Animal Genetic Resources,” commissioned by the FAO and funded by the Government of the United Kingdom of Great Britain and Northern Ireland, recognized that fundamental tension between the traditional ownership of AnGRs and new global conventions had emerged (Hiemstra et al., 2006) and this tension needs to be resolved on international level. Going through a number of options for AnGR regulation, tellingly the report ended with the summarizing paragraph claiming that “*[c]lassical ownership’ of AnGR includes physical ownership and communal ‘law of the land’ affecting livestock keeping and breeding. There is an increasing tension with developments in the realms of biodiversity law and intellectual property rights protection. Demarcation of these different rights systems and maintaining equity among different stakeholders is crucial to avoiding conflict and increased transaction costs. In this context, it is important to consider the rights of livestock keepers/breeders vis-à-vis national level sovereign rights, as well as obligations between patent holders and breeders/livestock keepers.*” (Hiemstra et al., 2006, p. 37).

The report had been commissioned as FAO wanted to clarify the options on how to navigate the world of new political and legal frameworks after CBD for AnGR management. 3 years later another expert report on AnGR raised a concern that relates to the fact that all different types ownership relations faced now a potential disruptive element. “*Private or communal ownership of AnGR, is potentially at least, challenged by national sovereignty over genetic resources. Individual owners may find that their rights to sell breeding animals or other genetic material, particularly across national boundaries, are restricted. Those seeking to buy specific AnGR may find that they are unable to do so, or that they can only do so on terms that are acceptable not only to the owner of the resources but also in compliance with national legislation*.”(Commission on Genetic Resources for Food and Agriculture, 2009, p. 29).

Finally, in November 2014, the Intergovernmental Technical Working Group on Animal Genetic Resources for Food and Agriculture concluded after its meeting that more work on AnGR is needed, but that at least the different types of *utilization* of AnGR, the criteria and ways in which the *country of origin* of AnGRs is assessed, and *access and benefit sharing policies* all need further clarification, although at global level a number of internationally binding legal treaties exist (Commission on Genetic Resources for Food and Agriculture, 2014, pp. 19–25).

For a long time, animals, breeds and their genetic resources were solely governed by rights that were based on physical access and use rights to animals as animals and breeds were seen as “wholes,” either as living animals or recorded breeds. A mix of private, semi-private and common ownership models for agricultural and farm animals have been in use, and these have also generated much discussion about the forms of entitlement over the life of the animals and the best possible ways to organize these relations (Hardin, 1968; David, 2011). However, as biotechnologies used in animal production have developed—increasing animal growth rates and carcass composition, enhancing disease resistance and improving hair and fiber production (Wilmut et al., 1992; Wheeler et al., 2010; Wheeler, 2013)—the value of individual farm animal, or even the value of a breed, is not only solely calculated in direct relation to its output of agricultural goods (e.g., meat, milk) but also by its value within the social system of breeding. Thus, farm animals and breeds are valuable also because their capacity to produce particular kind of offspring, or to transmit valuable features encoded within the DNA. This capacity can be either codified in rough ideas of maintaining a pure breed type or within the sophisticated algorithms calculating the Estimated Breeding Value in modern farms based on the development of the herdbook, an innovation that enables population management through exact recordings innovated in late 18th and early 19th centuries^4^.

Given that the two sources of value in farm animals have been recognized for over 200 years, it is surprising that at the present, in 2015, the global community dealing with AnGRs has ended up in a situation where the access, ownership rights and benefit sharing issues—issues that for a long time remained unchallenged—have become a matter of global concern and source of slowly proceeding political processes where there are no easy resolutions. The issue of “sovereignty” over all types of genetic resources described in the major international agreements gives the signatory states relatively free hands to develop and implement national laws and regulations. In fact, to fulfill their sovereignty over AnGRs, for example, the states must decide what types of entitlements and relationships over AnGRs they should implement, and how this relates to the national rights that farmers have over their animals, for example.

This paper presents two questions and two hypotheses on nature and status of the AnGRs in the post-CBD world:

(1) First, why are the issues of AnGRs for agriculture debated alongside more general issues of biological diversity;
  and
(2) Second, how should we understand that idea of sovereignty over genetic resources given to the signatory states of the CBD and the Nagoya Protocol in the context of AnGRs?

The first hypothesis builds on the cultural history of AnGRs movement in the political institutions, most notably in the FAO. I claim that early warnings about the need for the conservation and coordinated management of AnGRs for agriculture did not lead to action and resulted in a failure to mobilize larger communities to action. This, in turn, lead the animal geneticists affiliated with FAO and other interests parties to join forces with environmental conservation movement, especially United Nations’ Environmental Programme (UNEP), to gain international support to the issue of conservation then seen as a agenda priority.

Second, and following from the historical reason explained above, the way in which CBD and to certain extent the subsequent Nagoya Protocol defined and understood genetic resources owes much to the world of plant genetic resources (PGR). Defining the right and obligations of signatories through the PGR leads implicitly to the world of plant breeding, which operates differently from animal breeding practices, the key economic relations, and related biological processes. This is also why the key articles and provisions in the biodiversity conventions are couched in strong terms under national governments’ sovereign powers.

I claim that this has resulted in a world, where we have moved from a system where animals once were part of a seamless universal nature without political boundaries, to a world that is a collection of discrete “national genetic landscapes” safeguarded by state policies and legal provisions.

### The Short Institutional History of AnGR Concerns

The management of farm AnGRs become a topic immediately after the establishment of the United Nation’s Food and Agriculture Organization (FAO), however, the concerns related to genetic resources were first identified as a challenge for developing countries. The negative consequences of modern animal production aiming at increasing animal productivity started to raise doubts within the scientific community, and the first calls for genetic conservation followed quickly. Phillips, the first Deputy Director-General for the FAO remembers how he, as the first employeed of the animal section for FAO, had all his career “*already carried out activities relating to animal genetic resources…and the Organization’s involvement in this work dates back to 1946*” (Phillips, 1981, p. 5). From early on the worry was about losing local breeds to extinction in developing countries. Local animals were replaced with globally homogenized and more productive breeds that became easily available and were adopted at fast pace. Despite the early warning calls, little to no action aimed at conservation ensued at global level even if FAO produced a number of scientific reports and hosted a series of meetings around the issue between the early 1950s and 1960s.

It was only after the widespread negative impacts of Green Revolution became evident in the 1960’s that AnGRs became truly a global matter of concern also for scientists working within developed countries. This was the direct result of unmanaged use of new breeding techniques combined with shrinking and homogenized ecological habitats. For example, in the 1969 regional meeting of the European Association for Animal Production, the issue for “gene pool losses” was already clearly articulated by Maijala (1971) who also identified the root cause for these losses: “*The present era of frozen semen…has reactualized the problem of gene losses…The problem arises mainly from the fact that an effective utilization of the best animals of today automatically means setting aside the poorer animals, strains, breeds and even species*” (Maijala, 1971, pp. 403–444).

In response to these developments, FAO and the United Nations Environment Programme (UNEP) launched a joint project in 1974 with the title of “*Conservation of animal genetic resources*.” It had the key objective to “prepare a list of breeds of farm animals in danger of extinction together with an account of any measures which have been recommended or taken to prevent this extinction” (Mason, 1981, p. 17). A Consultation Report followed in Mason (1981) with a review of the work achieved by the project through the participating regional and national organizations, and made recommendations for future action.

This report was presented in a workshop for animal geneticists working with genetic resources and was framed with Phillip’s opening words, that simultaneously exhibited hope and exasperation on the current state of affairs. He proclaimed: “ *I am pleased to bid you welcome here, on behalf of the Director—General. It is indeed heartening to see such a distinguished group of animal geneticists assembled to consider the problems of identification, conservation and effective management of animal genetic resources. This is matter critical to man’s future, yet it has had little recognition and little real attention* (Phillips, 1981, p. 2).” Phillips’ opening speech betrays how, by the early 1980’s, the animal scientists had been awakened to the dire straits of genetic resources but the political support of the issue was still weak and more generally, unrecognized as an global political issue. It did not appear in the general global agendas as did other issues related to modernization and increase of production, such as the environmental movement which had started already in 1970s to attract more political attention and gained fast political weight in the international political arenas. As a result, the issue for farm AnGRs did not spur action nor attract funding for conservation efforts (Boyazoglu and Chupin, 1991).

Fast forward a decade into the early 1990’s, and one finds more explicit frustration toward the slow progress on conservation efforts and lack of coordinated international action. Explaining the issue and need for AnGRs conservation Hodges (1990), a Senior Officer at FAO wrote that “*the time for technical talk is over. The issues are clear. What is now needed is an effective international decision to provide funds to do what all agree is now necessary the global, regional and national levels*” (Hodges, 1990, p. 153). AnGRs needed more political support but this proved to be hard to gain without rethinking and reframing the issue, and joining forces with other institutional actors. International action did finally follow a few years later in 1992 when the FAO joined forces with the UNEP, and co-organized the Rio Earth Summit in Rio de Janeiro, Brazil. This was also the historical moment for AnGRs. This is the place and the time where genetic resources became newly articulated as parts of nature as they were linked directly to the recently introduced concept of “biodiversity,” the key theme of the global meeting for the world’s leaders.

In the meeting, UNEP and FAO introduced the global CBD, a convention aimed at saving biodiversity, for larger public and opened it for signatures. It was undersigned by some 160 countries at Rio de Janeiro and over 30 other countries followed suit during the upcoming years. Several of the Articles included in the Convention, addressed the issue of genetic resources directly and introduced an obligation to identify, report and take appropriate actions to conserve genetic resources. The long follow-up work finally resulted in The Global Plan of Action for Animal Genetic Resources (GPA), adopted in 2007, and the Guidelines on the Preparation of national strategies and action plans for AnGRs, published in 2009.

Given the half-century history of AnGR’s as a matter of concern for animal geneticists and long and idle wait for political action, the key question is why did the wide scale political traction to save genetic resources only emerge with the introduction of the CBD, leading to the global and national action plans and guidelines specific to AnGRs over a decade later?

## Early Failures in Valuation

The reason why the FAO and the regional institutions such as the EAAP failed in gaining political traction with their early alarms about the need for conservation measures relates to two shortcomings in the definition and the valuation of AnGRs.

First shortcoming was the lack of consensus in scientific definition, valuation and prioritization of AnGRs that would lead into simple and uniform action recommendations. The question on what is it exactly that needs to be conserved and how to prioritize the required conservation actions was left open, or at best was illustrated through a case of a few particular breeds. The second, and more important shortcoming was the failure of global political and legal identification of the responsible parties and beneficiaries of any value deriving from the costly conservation actions. This, in turn, relates to the fact that up until the CBD, AnGRs were treated as a mixture of “private” and “commons,” or as “club commons” (David, 2011) to be shared and used, subject only to individual farmers’ and breeding associations’ property right regimes and explicit regulations at country level.

After the introduction of the CBD the legal status of AnGRs changed globally as they were politically identified falling under the sovereign power of the signatory parties to the Convention—a major change and complication in access and benefit sharing relations that was later affirmed by the GPA in 2007 and later by the Nagoya Protocol. Understanding the latter is especially important as this understanding exposes the new overarching paradigm under which the value most of the AnGRs today are to be governed.

First, the failure to provide a clear direction for conservation relates to the arguments about the overall role of different kinds of AnGRs in animal production. When the concept of AnGRs were first introduced among the animal scientist, they were framed in and through two different ways (both scientifically informed) of demonstrating the role of AnGRs in animal production. The two ways—the “utilizationist” and “conservationist” standpoints—literally attributed the value of AnGRs in animal production in two incommensurable ways (and to some extent this debate still continues even today). Hodges (1984) report on genetic resources explained the main differences between the two approaches:

“*The utilizationist’s primary concern is the immediate usefulness of available genetic resources to improve livestock populations…The loss of breeds as distinct identities is not generally a concern, as long as the genes that make these breeds potentially useful are retained in the commercial stocks…The preservationist’s primary objective is long-term conservation of genetic resources for future use. This view emphasizes the value of preserving the widest possible spectrum of genetic diversity to be prepared for unpredictable changes of future needs. The greatest possible number of breeds are to be preserved as purebreds*.” (Hodges, 1984).

The differences of these two views boil down to conserving “the known useful genes” in one form or another versus conserving the “genetic diversity of whole animal breeds” to hedge the uncertainty deriving from unknown future needs. The first approach aims to save the sliced and diced, functionally valuable component of animals regardless of its “breed”; the other also the animal breeds in the purebred form and to maximize diversity as an insurance policy against future unknowns. Although analytically distinct from animals or breeds, the animal scientists first presented the issue of AnGR conservation as a choice between *isolated genetic components* immediately useful in the production of high performance animals or as the maximization of genetic diversity by the conservation of *local breeds in their animal forms*. In these two approaches, AnGR’s are conceptually presented as different objects of conservation and seen valuable for different purposes^5^.

Second, the failure to identify parties responsible for the conservation irked the conversations as this related directly to the economics of conservation, or more generally, to the political economy of global animal production. The problem was captured in a report produced by the United States’ board of Agriculture in its “Managing global livestock genetic diversity”:

*“The concept of conservation…is complex. One can think of live animals, being preserved in situ, or in some semi-artificial situation; alternatively one may think of cryogenic storage of sperm or fertilized ova or other tissues or gene segments. The economic problems are difficult with both live animals and with haploid or diploid cells. Who is to pay? There are also questions of how many to preserve, for how long, and where”* (National Academy of Science, 1993, p. 3).

Although plant varieties and their genetic material were protected by various intellectual property systems since 1930s (see Kloppenburg, 1988), AnGRs were used by farmers and breeder associations alike without generalized and specified rights or restrictions imposed at global level. Since there was no definition on the ownership rights over the genetic materials of animals, the global attribution of conservation responsibility through political processes proved to be impossible without more specific consideration. Yet, for pigs and chicken, ownership and responsibility questions have been more straightforward. This reflects what Tvedt et al., 2007, p. 8) note of the legal protection farm animals and their protection in general, and chicken and pig in particular:

“*For farm animals there are strong biological and physical means of protection available: The owner of the animal can more easily than the plant breeder have an overview and control over who is receiving genetic material from his animals or his population. For poultry and pig breeding, however, where farmers often buy hybrids whose genetics are more difficult to reproduce. The sale of hybrids is thus an important strategy for maintaining physical control over the genetic material by physical control over the material. For other breeds, in particular cattle, the physical ownership is often combined with a register, a herd book that maintains a protocol for the generations of animals fulfilling the criteria for registration.*”

This is why the different claims about the value of AnGRs and the need for their conservation, made by both the “utilizationists” and “conservationists,” rang to deaf ears outside the animal scientist circles. The failure to spur action was not based on the scientific challenge to demonstrate the value of AnGRs in animal production or the lack of consensus in setting the priorities of conservation. Instead, and above all, it was a problem of political economy: who is to pay? And even more importantly, who is to benefit?

## Global Re-framing of AnGRs

Food and Agriculture Organization remained active on AnGRs since the FAO/UNEP consultation program in 1980, established a Committee on Agriculture that kept reminding about the issue at the FAO Council level; designed a FAO expert consultation round on AnGRs in 1989 and in 1992 (Food and Agriculture Organization, 1990, 1999; Steane, 1992). What become clear over the years was that a global binding framework was needed.

Anticipating the global political agreement on AnGRs, de Haen (1992), Assistant Director-General of the Agriculture Department of FAO wrote in 1992 that “*it is clear that there is a greater awareness that a framework for the management of global animal genetic resources must be established. It is most appropriate that this Expert Consultation is taking place now in the context and timing of the Earth Summit, the United Nations Conference on Environment and Development (UNCED) to be held in Brazil in about eight weeks time*” (de Haen, 1992, p. 3).

The first reframing of the AnGR’s came in the form of the global CBD a few months later. The Convention had been long in preparation and FAO had been involved in its drafting phases influencing, among other issues, the inclusion of genetic resources to and their definition in the Convention text. There were two important re-framings in the Convention. First was the definition of the genetic resources, as genetic material of “actual or potential value” (CBD, Article 2). This definition bridged the two different views on the valuable material to conserve, or the “utilitizationist” and “preservationist” standpoints. Genetic resources become genetic material that could be attributed with demonstrable or imaginable value. But the question then arises: who has the right to attribute any value claims to AnGRs?

The other reframing answered to this question. Under the definitions of the Article 2 and the Article 15, genetic resources found “*in situ*” within the territory of a signatory were identified as belonging under the sovereign power of signatory states representing the nations of the world, reframing their ownership relationships globally. This is how CBD enacted an important political redefinition of genetic resources: previous problems in the definition of the value of nonhuman life were re-articulated through the politics of nationhood, in the idea of national differences found within the CBD’s vision of genetic nature.

With the convention, also AnGRs became tightly nested within the sovereignty of nation-states and their geography. A reversal of the old idea of nations being rooted in natural differences of human populations took place—nonhuman populations, conceptualized as “genetic resources,” could now be identified and placed under national or international jurisdiction in terms of their geographical location and the political powers that represented the nationhood that governed that geographical area. A global cartographic demarcation of nonhuman life took place as these novel objects of nature were grafted to the foundations of national sovereignty. They became a new part of the body of nations, a novel form of nonhuman nationhood.

The convention assumes significant amount of power over AnGR and their governance to signatory nation-states. Tvedt et al., 2007, p. 24) interpret the convention and its provisions in the following manner: “*The CBD presupposes the right of a country to exercise sovereign control over its AnGR (accompanied by a number of responsibilities). From the perspective of an exporting country, one of its main concerns is to maintain any property rights it may wish to retain over the AnGR after the resources have left the country. Similarly, it may wish to ensure that the rights of the exporter are respected by the buyer/importer of the AnGR. The most prominent rationale for a country to regulate export of AnGR would be to secure a right over that particular material in the future, including preventing that countries or companies gain control over these resources (e.g., through patenting or other forms of intellectual property rights), which might reduce the value of it in the exporting country.*”

This reframing introduced a whole new system where the value of any animal breed will be decided by the nations signatory to the parties but without any common reference what consists legitimate value claim over the material, except the condition of “*in situ*.” In the CBD, these are the “conditions where genetic resources exist within ecosystems and natural habitats, and, in the case of domesticated or cultivated species, in the surroundings where they have developed their distinctive properties.” (CBD Article 2). This “*in situ*” condition of valuable genetic resources has tremendous effects into how AnGRs are seen within the post-CBD world, especially as AnGRs were now removed from the idea of being freely circulated or tradable objects of nature. They stopped being global commons and instead become subject to the political powers of the Convention parties, many who did not have, and still today do not have, a clear stance what are “valuable” AnGRs to them, and how they will enact their sovereign powers over the access and benefit sharing to the valuable AnGRs. A definitional and legal disorientation followed.

The third re-framing of AnGR’s happened as they were presented through ideas derived from the plant and crop worlds. The FAO background study in the CBD on the “Exchange, Use and Conservation of Animal Genetic Resources” acknowledged this as a major problem. It explained:

“*[a]lthough current debates regarding agricultural genetic resources have largely had a crop/plant focus, these discussions, and the international instruments or agreements that are emerging have tended to frame the debate for AnGR as well. At first sight plant breeding does not differ much from animal breeding. The genetics of plants and animals are based on the same principles. Plant and animal breeders both need genetic diversity in order to advance and the genetics determine adaptation to particular agro-ecological circumstances, as well as product qualities to a large extent. However, plant varieties can be protected by plant breeder’s rights (UPOV), which is not the case for animal breeds/strains. Plant breeders aim at the development of new uniform varieties that are defined by certain phenotypic traits that can identify them from other varieties. Farm animal breeding is largely based on the selection of individuals within populations rather than selection between populations or strains. Farm animal breeders are interested in individual animals (within populations/breeds), while the whole population of a plant variety (clones) is the main focus of plant breeders*.” (Hiemstra et al., 2006, p. 22).

The third reframing, then, pointed to the difference of animal and plant genetic resources as biological bred resource and legal protected asset: animals might carry interesting genetic traits but it is difficult to exploit one unique genetic characteristic, there are no large international breeding centers but most breeding happens in farms—except for poultry and partly for pigs—and the centers of origin or diversity for AnGR are not as clearly defined as for plants. Most importantly, farmers are not protected by internationally binding rights frameworks—plant breeders, however, are by the International Union for the Protection of New Varieties of plants (UPOV)^6^. The differences between plant and AnGRs make it hard to enforce only system for the two, however, the CBD does exactly this by enforcing the sovereignty of the signatory states as its starting point for rights and obligations via the discourses mostly appropriate to plant genetic resources.

These re-framings of the AnGR dictate much of how global action now unfolds. 15 years after the CBD, in 2007, the state representatives adopted the first “Global Plan of Action for Animal Genetic Resources” (GPA) at the Interlaken Conference held in Switzerland, something that was called a “historical breakthrough” by the FAO Director General Jacques Diouf (Food and Agriculture Organization, 2007, p. iii). The GPA includes the “Interlaken Declaration on Animal Genetic Resources,” in which the sovereign rights of states over their AnGRs for food and agriculture was restated (declaration point 2).

### *In situ*, Transboundary, and Domestic Applications

The fact that animals can move across politically established boundaries created a potential problem to these sovereign rights, however, and led to new politically innovated categories of AnGRs, such as “transboundary” species for criss-cross institutionalized country borders. The GPA explained: “Assessing the status of animal genetic resources on a global scale presents some methodo-logical difficulties. In the past, analysis of the Global Databank to identify breeds that are globally at risk was hampered by the structure of the system, which is based on breed populations at the national level. To address this problem…a new breed classification system was developed. Breeds are now classified as either local or transboundary, and further as regional or international transboundary” (Food and Agriculture Organization, 2007, p. 13).

With these political documents not only did animals considered as genetic resources become “national” pertaining to a state, but some of them also became “transboundary,” regionally and internationally. The result of this is that political categories infuse with conservation science categories because of the political economy involved in the ownership rights over the actually or potentially valuable genetic resources.

These categories are as much politically informed as they are scientifically true. The definitions of “*in situ*” or “transboundary” are inherently related to the political cartographic demarcation of the natural ecologies of domesticated animals, pointing to the deep connection between politics of value and the science of conservation of farm genetic animal resources. This is what eventually created the incentive for nation-states to act upon the issue of genetic erosion of animal populations, but is now, at the same token, generating new challenges that are beyond the scope of animal scientists or even international organizations to solve.

This complexity is reflected on how the national legislations have been drafted and implemented. Writing about the challenges in the implementation of the CBD legal experts Buck and Hamilton claim that “*[t]he complex subject matter of ABS, its potentially far reaching impact on uses of genetic resources and related information as well as the lack of detail in Articles…have all combined to result in a very low level of domestic implementation by Contracting Parties to the CBD. By 2007, only 39 of the then 189 Contracting Parties had established domestic legislation or were in the process of doing so*.” (Buck and Hamilton 2011, p. 48).

Reporting back on the negotiations of the Nagoya Protocol in Japan in 2010, the protocol that is to meant to clarify the initial CBD, they point out that the key to really “understanding” the real effects of the CBD and Nagoya is dependent on how national governments use their sovereign powers: “*The adoption of the Nagoya Protocol was a major achievement in international biodiversity policy making in 2010…Further international work preparing the entry into force of the Nagoya Protocol will be needed. However, most efforts over the coming years will need to be at domestic level, developing implementing rules to prepare ratification. In all Parties with well-developed or emerging research and development systems this will require significant awareness-raising with stakeholders from research and industry and will result in quite some discussions*.” (Buck and Hamilton, 2011, p. 60).

Most importantly, the national implementation has to take into account that access should take place on “mutually agreed terms” and “be subject to prior informed consent,” conditions found in the original CBD and all subsequent treaties. However, other aspects of AnGR can also be regulated, and some of the countries have enacted already requirements for animal genetic material import and export. FAO’s Technical Working Group on AnGR Access and Benefit sharing issues explained in its recent report in 2014 that “*[t]he sovereign right of states to determine access to genetic resources should not be confused with other categories of entitlement, such as the private ownership of an animal. A farmer’s ownership of an animal may be conditioned by certain laws. For example, animal welfare legislation may regulate the handling, husbandry and transport of the animal. Other laws may require the animal to be vaccinated against specific diseases, and so on. In a similar way, ABS measures may require that, even though an animal is the private property of a farmer or the collective property of a community, certain conditions (e.g., related to the need for “prior informed consent”) must be met before it can be provided to a third party for research and development*” (Commission on Genetic Resources for Food and Agriculture, 2014, Item 18).

Indeed, some of the countries have already exercised their sovereign rights. For example China has adopted a set of rules to AnGR, or “*Measures of examination and approval of the entry and exit of animal genetic resources and the research in cooperation with foreign entities in their utilization*,” in 2008. These include a set of import and export rules, such as prohibition “*on the export of newly discovered and unverified*” AnGR in cooperation with “*any foreign institution of individual*.” Also, any research and use of AnGR involving foreign collaborators requires permission from the Chinese authorities. South-Africa, on the other hand, now requires a “*genetic impact assessment*” before the import of new breeds. These studies need to be prepared by reputable South African animal scientists and submitted to the relevant authorities (see Commission on Genetic Resources for Food and Agriculture, 2009, p. 34). The national implementation of the sovereign rights over genetic resources can happen in many ways, not only by regulating of access or benefit sharing but also by use and impact, as the examples from China and South Africa demonstrate.

## Conclusion

The challenges are located now within the realm of national politics where the *in situ* condition of genetic resources are turning animals into collections of nationally valuable animals, governed not by the previous ideals of global commons but by the logic of “actual and potential” value, by innovated political re-categorizations of natural beings, and by national restrictions to the access, use and benefit sharing of AnGRs. We have moved from a world where animals once were part of a seamless universal nature without boundaries to a world that is a collection of discrete “national genetic landscapes.”

Over the course of the short history of AnGR conservation, the natural identities of farm animals have been refashioned from being objects of breeding to boost the productivity of individual animals and breeds to objects that can be defined actually or potentially valuable as nationally recognized genetic resources. The change in their identity is a creative outcome product of the animal breeding and conservation sciences that have argued the value of animals on the basis of scientific evidence as well as the global politics surrounding the ownership rights over genetic resources considered valuable. AnGRs, including farm animals, are now as much political as they are scientific, as much “cultural” than they are “natural” by their essence.

Table 1 above summarizes the key changes in the conceptualization and valuation of AnGRs before and after the introduction and ratification of the CBD, the Global Plan of Action and the Nagoya Protocol. What becomes clear while looking at the key changes in the value system of AnGRs is that AnGRs have become increasingly complex objects for breeders, scientists and politicians alike, with no easy answers to how the balancing of rights, responsibilities and benefit sharing in the near future. While AnGRs have finally become a global issue with high political priority and action, so have the political conditions under which the animals live become inherently global entanglement of science and politics, culture and nature.

**TABLE 1:** **The historical changes in the value system for animal genetic resources**.

**Animal genetic resources**	**Pre-CBD/GPA/Nagoya**	**Post-CBD/GPA/Nagoya**
Nature of animal genetic resources	• Natural breeds or functional genetic components • Identification based on scientific definition and evaluation of conservation need	• Natural-Cultural objects • Identification based on the politically agreed “*in situ*” condition • Genetic material found within the geographically bounded territories of the nation-states, with the exception of politically innovated “transboundary” conditions and categories of animals
Access and benefit sharing	• Private access, or club commons • Freely usable for those who have physical access and local permission to use • No enforced benefit sharing	• National sovereignty to decide • Usable only under the rule of the sovereign party to the conventions • Local political decision on access and benefit sharing principles.
Criteria for valuation	• Actual or potential value, no consensus in general	• Actual AND potential value • Based on territorial *in situ* condition and local political valuation of important national genetic landscapes to conserve

At the same time, the status of AnGRs that reside outside of the CBD system—either owned by private companies or breeding societies before the entry into force of the CBD in 1993—is unclear. Although they are not objects of CBD’s articles, they might still be affected by and become targets of legal interventions by way in which for example China and South Africa have applied the sovereignty over genetic resources within their respective AnGR regulations. This makes the global system even more complicated, and most likely with a number of unforeseen challenging cases in the future.

The CBD, the Global Plan of Action and the Nagoya Protocol present a global value system framing AnGRs in a way that is finally generating conservation action at national level. But on the global level, the system is more muddled than ever calling for a great deal of conceptual, political and legal analysis to bring more clarity to the current condition that requires the generation of discrete genetic landscapes and marks AnGRs with their nationally correct *in situ* location as their political condition of existence. Given the complex history of AnGRs as a global matter of concern, creating clarity to the present situation will not be easy.

At least three key questions need to be clarified with regards to AnGR and the different claims laid over them in order to move on in the global politics, in the creation and implementation of legal frameworks at national level, and in the reflection of the true impact of the CBD and Nagoya Protocol.

(1) What is the true scope of the CBD and Nagoya Protocol in terms of AnGR types? Are there types of AnGRs that remain totally unaffected by and reside outside of the scope of the global conventions?
(2) Do the signatory parties (nation-states) have prototypical reactions to—or at least broadly identifiable patterns in—the implementation of their sovereign powers over AnGR?
(3) If the signatory parties do exhibit identifiable patterns, a guiding typology of CBD and Nagoya Protocol implementation at national level would help to make sense of how governments are adopting the global agreements (e.g., types of entitlement claims, access regulations etc) at large.

Addressing these three points would already give a much richer and much more coherent overview of AnGRs’ status in the post-CBD and post-Nagoya Protocol world than is currently available for public. We do suggest that the institutions driving the global framework on genetic resources provide it soon.

### Conflict of Interest Statement

The author declares that the research was conducted in the absence of any commercial or financial relationships that could be construed as a potential conflict of interest.

## Footnotes

1 https://www.cbd.int/

2 http://www.cbd.int/abs/

3 The names of these expert bodies are: the Intergovernmental Technical Working Group on Animal Genetic Resources for Food Agriculture, the Intergovernmental Technical Working Group on Plant Genetic Resources for Food and Agriculture to deal with specific matters in their areas of expertise, and the Intergovernmental Technical Working Group on Forest Genetic Resources.

4 General herdbooks emerged in Europe in the late eighteenth—and early nineteenth century—the first one for cattle was the “*Short-Horned Cattle Herd*” book, published in 1822 in England. Elsewhere, general registries were published in France (1855), Germany (1864), Holland (1874), and Denmark (1881) (Derry, 2003, p. 8; Walton, 1999, p. 153; Ritvo, 1995, p. 420). The idea of “Estimated Breeding Value” is based on this herdbook keeping but introduces a more refined statistical modeling into the calculation of breeding value of a individual animal at the end of C21th.

5 There are a number of ways to maximize diversity. Conserving a sum of isolates of pure inbred populations will allow saving rare genetic combinations adapted to specific environmental conditions but might result in losing overall diversity. Other options, such as maintaining a large out bred population resulting from crossbreeding, would also provide a large diversity but is not usually the overall aim of conservation programs. It is today generally recognized that a combination of ex-situ and in-situ measures are complementary strategies.

6 http://www.upov.int
